# Effects of habitat homogenisation on assemblages associated with mussel clumps

**DOI:** 10.1371/journal.pone.0269308

**Published:** 2022-06-01

**Authors:** Puri Veiga, Juan Moreira, Leandro Sampaio, Jesús S. Troncoso, Marcos Rubal

**Affiliations:** 1 Interdisciplinary Centre of Marine and Environmental Research (CIIMAR) of the University of Porto, Matosinhos, Portugal; 2 Departamento de Biología (Unidad de Zoología) & Centro de Investigación en Biodiversidad y Cambio Global (CIBC-UAM), Universidad Autónoma de Madrid, Madrid, Spain; 3 Centro de Investigación Mariña, Departamento de Ecoloxía e Bioloxía Animal, Laboratorio de Ecoloxía Costeira (ECOCOST), Universidade de Vigo, Vigo, Spain; Maurice Lamontagne Institute, CANADA

## Abstract

Biodiversity loss is considered one of the main threats to marine ecosystems. In this framework of biodiversity decline, organisms that provide biogenic habitat play a relevant role by their capacity to structure assemblages and influence ecological processes. The Mediterranean mussel *Mytilus galloprovincialis* is considered an ecosystem engineer because it alters local environmental conditions maintaining habitat suitability for other organisms, and enhancing local biodiversity. Although it is widely recognized that mussel beds increase diversity, the drivers shaping these assemblages are poorly explored. We investigate whether mussel size homogenisation shapes the abundance, richness and structure of macrobenthic assemblages associated with mussel beds in two shores of the Galician coast (NW Spain). At each shore, two sites, 10 m apart, were selected and at each site, faunal assemblages were compared between mussel clumps showing shells of various sizes (control), and mussel clumps with closely similar-sized mussels, considered as homogenised. Homogenised clumps showed, in general, higher values in total number of individuals and species than control clumps. Regarding the effect of mussel size homogenisation on the multivariate structure of the assemblages, significant differences between control and homogenised clumps were found in three out of the four sites. Most relevant associated species usually reached higher abundances in homogenised clumps than control ones. Therefore, mussel size homogenisation influenced the structure of the macrofaunal assemblages associated with mussel beds but, its effect was context dependent (i.e., varied with sites). Information about the species contribution to dissimilarities among homogenised and control clumps was provided and the potential influence of sediment and algae on mussel clumps was discussed.

## Introduction

Current rates of biodiversity loss surpass historical background levels by several orders of magnitude, mainly because of land use modification, climate change, invasive species, overexploitation and pollution [1, 2]. Credible scenarios for the near future reveal an increasing trend for biodiversity loss in the next 50 years [1]. Moreover, the incomplete taxonomic knowledge, particularly in marine systems, surely leads to predictions that underestimate species extinction rates, as many species could become extinct before being described [3, 4]. Loss of biodiversity undermines ecosystems’ abilities to function efficiently and thus weakens their capacity to provide society with a number of relevant goods and services [5, 6]. This is particularly crucial in the context of the current changing climate scenario because the removal of biodiversity also reduces nature’s resilience to change [7]. Therefore, rates of biodiversity decline are now considered one of the main stressors impacting marine ecosystems [7, 8].

In this biodiversity loss framework, marine organisms that provide biogenic habitat play a relevant role by their capacity to structure assemblages and influence ecological processes [9]. Many of them (e.g., canopy macroalgae, corals and mussels) are considered ecosystem engineers because they alter local environmental conditions maintaining useful habitat for other organisms, thus enhancing local biodiversity [e.g., 9–12]. The Mediterranean mussel *Mytilus galloprovincialis* Lamarck, 1819 is an important economic marine resource and very abundant along the south Atlantic European and Mediterranean coasts, where this species is native [13, 14]. It is a gregarious species that attaches to hard substrates using byssal threads and establishes dense clumps or beds on exposed or moderately exposed temperate rocky shores, such as in the northwest portion of the Iberian Peninsula, where temperature and food supply are optimal for the species [14]. In areas where mussel species are dominant, they can prevent the attachment of other sessile organisms by competing for primary substratum [9, 12]. However, mussels, as ecosystem engineers [15, 16], modify the local environment and provide secondary substratum and structurally complex three-dimensional habitat for many species [e.g., 9–12, 17, 18]. Consequently, the habitat provided by shells of live and dead mussels and the interlacing byssal threads ameliorates physical and physiological stress and provides protection against removal by predators or dislodging by waves [15]. Moreover, the metabolic activity of mussels also influences faunal assemblages [19]. For instance, sedimentation rates rise due to the biodeposition of organically enriched faeces and pseudo-faeces that, in turn, constitute an important food supply for deposit feeders [20], allowing for the settlement of infaunal organisms [17] and, being nutrient-rich, promote the growth of marine algae [21].

Although it is widely recognized that mussel beds increase diversity [e.g., 9–12, 17, 18], the drivers shaping these assemblages are poorly explored [10, 17]. Additionally, studies that have assessed the relationship between mussel population structure (i.e., density, size and age) and abundance, composition or diversity of mussel-associated assemblages have shown contrasting results [22]. Some works [e.g., 12, 13, 23] reported a negative relationship between mussel abundance and diversity of assemblages, whereas others have found a positive relationship between mussel abundance and the number of species and abundance of fauna [24] or no relationship at all [22]. On the other hand, Hammond [25] and Hodgson et al. [12] showed that there was no relationship between mussel size and species richness and diversity, whereas Cole and McQuaid [24] found a negative relationship between mussel size and the number of species and the abundance of fauna. However, a higher diversity was found among larger but older mussels [26], thus being unable to separate age and size effects [17]. The study by O’Connor and Crowe [17] showed that differences in mussel size shape the abundance and proportion of individuals, but not diversity, yet the effect of mussel size depends on location (i.e., significant differences were only detected in one of the two studied localities). Therefore, a basic understanding of how individual traits of ecosystem engineers influence biodiversity is still necessary [27].

*Mytilus galloprovincialis* is mainly cultured on rafts along the north-western Iberian Peninsula, which is the most important mussel producing area in the EU [28]. Although *M*. *galloprovincialis* reaches high abundances in this region [14], wild populations are severely exploited since most of the mussel seed used in farming is collected from natural populations [28]. In addition to harvesting, mussel beds also suffer the effects of other anthropogenic disturbances, such as acidification, pollution and urbanization [29–35] as well as being submitted to natural disturbances (e.g., predation, wave exposure) [36, 37]. Mussel clumps in natural systems usually cluster in different mussel sizes, however, they can also display a very similar size, probably as a consequence of harvesting or other anthropogenic or natural disturbances that previously wiped out the mussel clumps and allowing new settlement of mussel recruits. This homogenisation of mussel size could influence the vagile faunal assemblages harboured by mussel beds but this topic has not yet been investigated.

The present study aimed to investigate the role of habitat homogenisation in shaping the abundance, richness, and structure of vagile faunal assemblages associated with mussel beds on two shores of the Galician coast where harvesting for mussel seed is intense. We expect that differences in habitat structure determined by differences in mussel size structure may affect the diversity of assemblages on mussel beds, i.e., beds composed by mussels of different sizes will provide a more heterogeneous habitat than those homogenised (mussels of similar size) and therefore the later will support fewer species.

## Material and methods

### Study area

This study was done in two intertidal rocky shores located in the external area of two Galician rias (NW coast of Spain): Ría de Muros (42°45’19.88"N, 9° 6’21.06"W) and Ría de Vigo (42° 5’37.95"N, 8°53’39.81"W). Galician coastline constitutes the northern limit of the North Atlantic Upwelling System, one of the world’s major upwelling zones, and therefore is a highly productive area [38]. This area presents a semidiurnal tidal regime, with the largest spring tides of 3.5–4.0 m [40]. The coast is in general exposed to the long swells from the NW and W, and to the SW storm waves [39]. The mean wave height differs strongly among seasons. In the spring-summer period, wave heights usually range between 1 and 3 m. Most storms happen during autumn–winter months (October–March) when wave heights typically surpass 7 m [40]. Rocky shores are typically granitic rocks and present a significant slope, >40% and with maximal values of 60%.

### Sampling and sample processing

Biodiversity associated with two different kinds of *M*. *galloprovincialis* clumps (control *versus* homogenised), hereafter called clump kind (Ck), at different spatial scales was assessed. Clumps showing mussels with a wide range of sizes were considered as control and clumps showing closely similar-sized mussels were considered as homogenised. Two rocky shores (Sh), where mussels are intensively harvested (Muros: M and Oia: O), were selected in Galicia (NW Iberian Peninsula) separated about 80 km. At each rocky shore, two sites (Si) were randomly selected (about 10 m apart) at mid intertidal level (between 1.5 m and 2 m above Chart Datum). Four replicates per clump kind were randomly collected at each site and shore by scraping off all mussels and associated fauna from within 10 x 10 cm quadrats. To avoid border effects, quadrats were collected at the centre of mussel beds bigger than 20 x 20 cm. Sampling was done in November 2018. Samples were stored in labelled plastic bags and frozen for further processing. In the laboratory, the number of mussels from each replicate was counted to estimate mussel density. Moreover, as measuring every mussel from each replicate was practically impossible due to the large numbers occurring in each quadrat, shell length was measured for 20 mussels per quadrat, using a calliper (±0.1 mm). Thus, mussels from each replicate were randomly placed in a tray. The centre of the tray was marked with a cross and mussels were selected following concentric circles from the centre of the tray up to 20.

Clumps were washed in freshwater and shaken vigorously several times to remove vagile fauna; water was then sieved (0.5-mm mesh size) to retain the macrofauna, that was conserved in formaldehyde, and specimens were sorted, identified to the lowest possible taxon (usually species level) and counted.

### Data analyses

All univariate and multivariate data were analysed using a three-way mixed model design with Clump kind (Ck) as a fixed orthogonal factor with two levels (Control and Homogenised), Shore as a random orthogonal factor with two levels (Muros and Oia) and Site as a random factor nested in shore with two levels (site 1 and site 2) and four replicates per clump kind.

Analyses of variance (ANOVA) were done to test for differences between clump kinds on mussel density, mean size and the standard deviation of mussel size; the latter was considered a proxy for habitat homogenisation. ANOVA was also used to test for differences on the total number of individuals (N) and total number of taxa (S) of macrofauna associated with mussels in control and homogenised clumps. Cochran’s C tests were previously done to check for homogeneity of variances, and when the test was significant (p<0.05) data were Ln(x+1) transformed to remove heterogeneity. When ANOVA indicated significant differences (p<0.05) between clump kinds, a post hoc Student-Newman-Keels (SNK) test was done to explore differences between homogenised and control clumps.

Permutational multivariate analysis of variance (PERMANOVA) [41] based on a Bray-Curtis untransformed dissimilarity matrix was used to analyse the multivariate structure of macrobenthic assemblages associated with mussel clumps. When PERMANOVA showed significant differences (p<0.05), a pair-wise comparison (999 permutations) was done to explore differences among all pairs of levels of the selected factor. As the number of unique permutations for the pair-wise comparison was low, Monte Carlo P-values were considered [41].

In order to test whether differences of assemblages between clump kinds were due to different multivariate dispersion between groups rather than in the location of centroids, the PERMDISP procedure was used [42]. Multivariate patterns were illustrated by non-metric multidimensional scaling (nMDS) ordination based on centroids for the Ck x Si interaction.

The SIMPER procedure [43] was used to determine the contribution (δi%) of each taxon to the Bray-Curtis dissimilarity between assemblages associated with control and homogenised mussel clumps (δi). A taxon was considered important if its contribution to the total percentage dissimilarity was >4%. The ratio δi/SD(δi) was used to quantify the consistency of the contribution of a particular taxon to the average dissimilarity in all pair-wise comparisons of samples between control and homogenised clumps. Values ≥1 indicated a high degree of consistency [44].

## Results

### Habitat provided by mussel clumps

**Fig 1:**
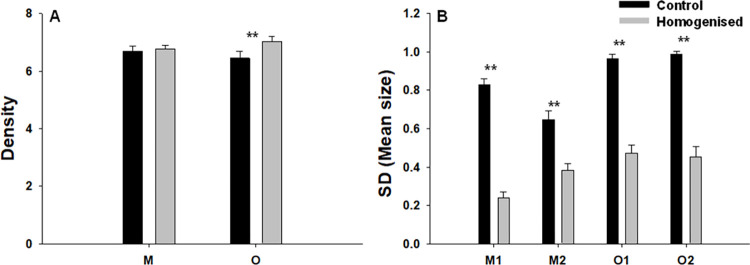
Density of mussels and habitat homogenisation. Mean values (+SE) of density (number of mussels per 10 cm^2^; Ln(X+1) among locations (sites pooled) (A) and standard deviation of mean size for each site (B) of *Mytilus galloprovincialis*. Asterisk indicates significant differences (SNK tests, **: p<0.01).

Mussel density varied significantly with respect to the Ck x Sh interaction (Table 1). In Muros, mussel density was similar between control and homogenised clumps, whereas in Oia homogenised clumps showed significantly higher densities than control clumps (Fig 1A). Regarding shell length of mussels, the following size ranges were found: Muros1 (0.6–4.7 cm in control and 0.6–1.9 in homogenised clumps); Muros2 (0.4–3.1 in control and 0.3–1.9 in homogenised clumps); Oia1 (0.4–3.9 in control and 0.6–2.4 in homogenised clumps); Oia2 (0.4–4 in control and 0.6–2.4 in homogenised clumps). Mean size of mussels did not vary significantly between control and homogenised clumps (Table 1). In contrast, the standard deviation of mean mussel size varied significantly as a function of the Ck x Si interaction (Table 1). Mussel size was significantly more heterogenous in the control clumps but the magnitude of these differences differed between the two studied shores (Fig 1B).

**Table 1:** Summary of ANOVAs testing differences on density, mean size and standard deviation of mean size of *Mytilus galloprovincialis* between clump kinds.

Source of variation	df	Density		Mean size		SD of Mean size	
		MS	*F*	MS	*F*	MS	*F*
Clump kind	1	0.8390	1.74	1.3799	38.93	1.7584	120.10
Shore	1	0.0001	0.00	0.3949	3.64	0.3002	420.26**
Site (Sh)	2	0.9633	4.26*	0.1086	3.77*	0.0007	0.14
Ck x Sh	1	0.4822	**39.87** *	0.0354	0.83	0.0146	0.27
Ck x Si (Sh)	2	0.0121	0.05	0.0429	1.49	0.0540	**10.51** ^***^
Residual	24	0.2259		0.0288		0.0051	
Total	31						
Transformation		Lx(x+1)		none		none	
Cochran’s test		*C* = 0.32	ns	*C* = 0.29	ns	*C* = 0.28	ns

Ck: Clump kind; Sh: Shore; Si: Site; df: degrees of freedom; MS: mean squares; *F*: F-ratio; ns: not significant

*: p<0.05

**: p<0.01

***: p<0.001.Relevant significant differences (i.e., including fixed factors) are indicated in bold.

### Macrobenthic assemblages associated with mussel clumps

A total of 14 443 individuals (9 953 in homogenised and 4 480 in control clumps) and 115 taxa (103 in homogenised and 73 in control clumps) were found. Molluscs accounted for 39% of the total individuals, followed by arthropods (27%), nematodes (21%) and annelids (11%). The numerically dominant taxa below phylum were the bivalve *Lasaea rubra* (23% of total individuals), oligochaetes (8%), the amphipods *Hyale* spp. (8%) and *Stenothoe monoculoides* (4.5%), and the gastropods *Rissoa parva* (5%) and *Odostomia scalaris* (4.2%). The remaining taxa each accounted for less than 4% of the total abundance observed. Forty-one species were exclusively associated with homogenised clumps whereas 11 species were only found in control clumps.

**Fig 2:**
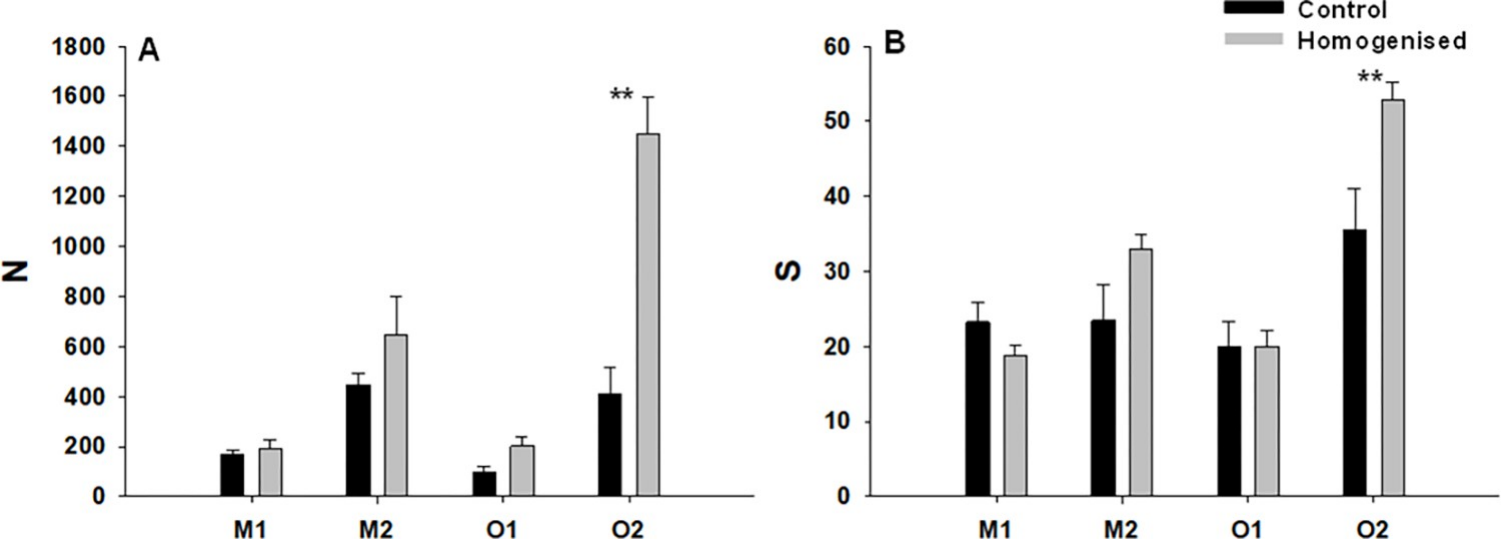
Abundance and richness of macrofauna associated with *Mytilus galloprovincialis*. Mean values (+SE) of total number of individuals (A) and total number of taxa (B). Asterisk indicates significant differences (SNK test, **: p<0.01).

Total number of individuals (N) and total number of taxa (S) varied significantly with respect to the Ck x Si interaction (Table 2). N and S showed significantly higher values in homogenised clumps compared to control clumps but only in site O2 (Fig 2A and 2B).

**Table 2:** Summary of ANOVAs evaluating the variation in the total number of individuals (N) and total number of taxa (S) between clump kinds.

Source of variation	N			S	
	df	MS	*F*	MS	*F*
Clump kind	1	936054.0313	2.24	247.5313	3.30
Shore	1	251163.2813	0.17	442.5313	0.35
Site (Sh)	2	1488980.4063	48.56***	1269.1563	29.34***
Ck x Sh	1	418383.7813	0.93	75.0313	0.30
Ck x Si (Sh)	2	447782.6563	**14.60** ***	246.7813	**5.70** ^**^
Residual	24	30662.1563		43.2604	
Total	31				
Transformation		none		none	
Cochran’s test		*C* = 0.37	ns	*C* = 0.40	ns

Ck: Clump kind; Sh: Shore; Si: Site; df: degrees of freedom; MS: mean squares; *F*: F-ratio; ns: not significant

**: p<0.01

*** p<0.001. Relevant significant differences (i.e., including fixed factors) are indicated in bold.

**Fig 3:**
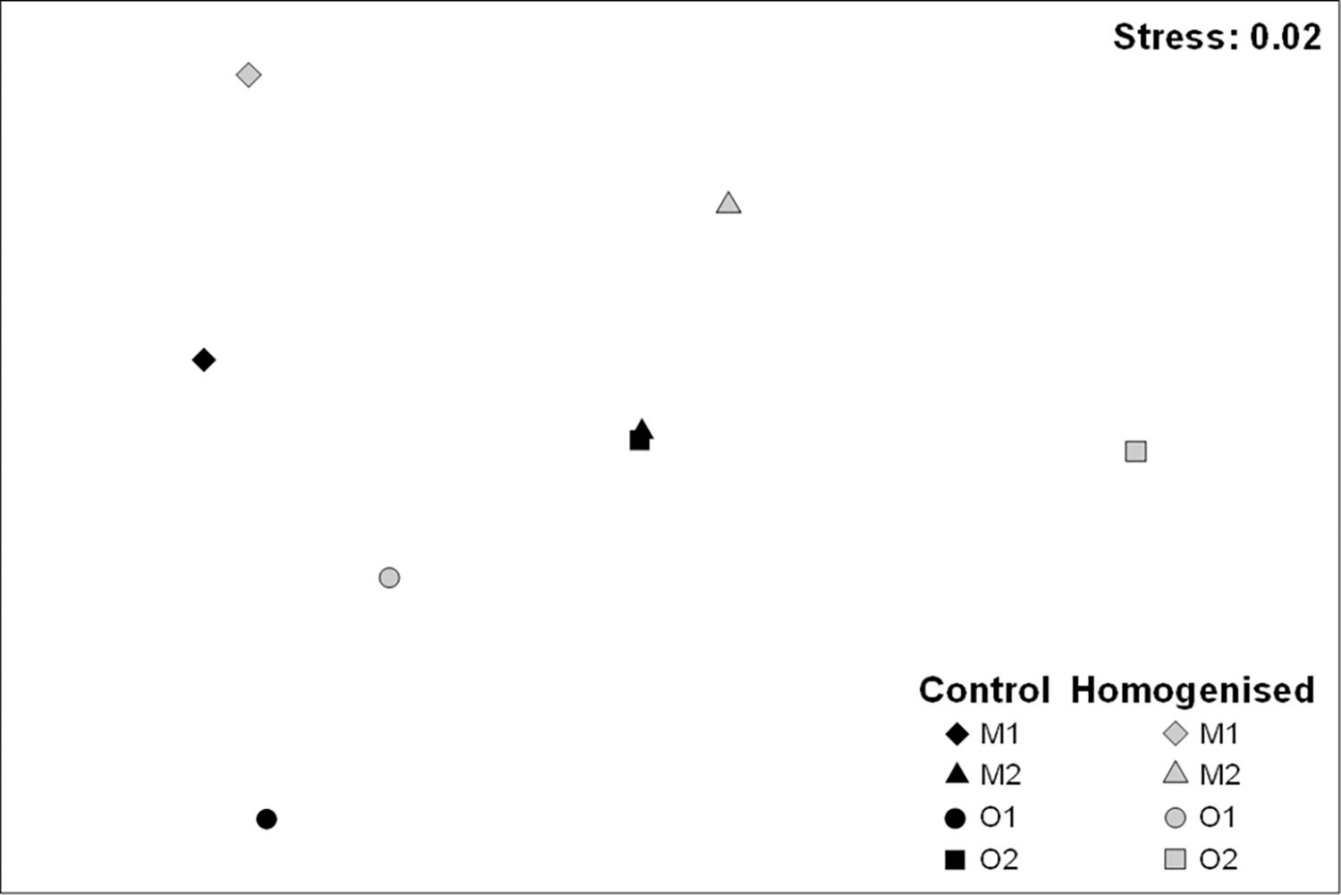
nMDS plots of centroids from control and homogenised clumps at four studied sites. Based on centroids for the Ck x Si interaction.

The structure of associated assemblages also showed significant differences for Ck x Si (Table 3). The nMDS ordination showed a clear separation between homogenised and control clumps, with the exception of M2 where pair-wise test did not detect differences (Fig 3, Table 3). Moreover, the PERMDISP analysis for the interaction Ck x Si (F = 1.08, p = 0.726) indicated that the dispersion of replicates did not provide a significant contribution to the observed differences between the structure of the assemblage associated with homogenised and control clumps.

**Table 3:** Results of PERMANOVA testing differences in the structure of the macrobenthic assemblage between clump kinds. Analyses based on matrix of Bray-Curtis dissimilarities from untransformed data. Monte Carlo p-values were considered because the number of unique permutations was low.

Source of variation	df	MS	*Pseudo-F*
Clump kind	1	4872.4	1.3579
Shore	1	7063.9	0.58414
Site (Sh)	2	12093	10.618**
Ck x Sh	1	3588.1	0.94363
Ck x Si (Sh)	2	3802.4	**3.3388** **
Residual	24	1138.9	
Total	31		
**Pair-wise test**		**t**	
M1		**2.0168** ^*^	
M2		0.259	
O1		**1.8819** ^*^	
O2		**2.6687** ^**^	

Ck: Clump kind; Sh: Shore; Si: Site; df: degrees of freedom; MS: mean squares

*: p<0.05

**: p<0.01. Relevant significant differences (i.e., including fixed factors) are indicated in bold.

SIMPER analysis identified 32 taxa that contributed most to differences between control and homogenised mussel clumps. Collectively, these taxa contributed more than 90% to the total dissimilarity (S1 Table). The contribution to the percentage of dissimilarity of *Hyale* juveniles, Nematoda, *Hyale nilssoni/stebbingi*, *Lasaea rubra*, *Odostomia scalaris*, Chironomidae and *Jaera albifrons* was consistent among pair-wise comparisons in the three sites (M1, O1 and O2) which showed significant differences on the structure of macrofauna associated with control and homogenised clumps (S1 Table). *Rissoa parva*, *Skeneopsis planorbis*, *Gibbula umbilicalis*, *Stenothoe monoculoides* and *Barleia unifasciata* were only responsible for differences between homogenised and control clumps in M1 and O2; Harpacticoida in O1 and O2; and Nemertea and *Idotea pelagica* in M1 and O1 (S1 Table). Other taxa were responsible for the differences between control and homogenised clumps in only a single site, namely *Nucella lapillus* and Dolichopodidae in M1, *Patella depressa* in O1 and Oligochaeta in O2.

Most taxa showed higher abundance in homogenised clumps compared to the control ones (S1 Table). However, *G*. *umbilicalis* and *I*. *pelagica* were more abundant in the control clumps. Moreover, five taxa in M1 (i.e., *Hyale* juveniles, *H*. *nilssoni/stebbingi*, *J*. *albifrons*, *S*. *monoculoides*, *N*. *lapillus*) and Chironomidae in O1 were also more abundant in the control clumps (S1 Table).

## Discussion

Mussel size homogenisation influenced the structure of the macrofaunal assemblages associated with mussel beds although its effect was context dependent. Similar results were found by O’Connor and Crowe [17] who showed that the mussel size effect on the assemblages’ structure varied spatially. Moreover, Cole and McQuaid [24] suggested that regional effects are more relevant than habitat structure in shaping the fauna associated with mussels. Likewise, Sardinha et al. [45] found that facilitation by the golden mussel *Limnoperna fortunei* is highly site-specific and Çinar et al. [11] pointed out that structure of the faunal assemblages in *M*. *galloprovincialis* beds is determined by local environmental conditions. Our results also supported this because the effect of clump kind on the structure of associated assemblages with *M*. *galloprovincialis* was dependent on site. Thus, the scale of site seems to have more influence than the larger considered scale (shore), in agreement with previous studies that have found more variability at smaller scales [46].

According to Hodgson et al. [12], the composition, abundance and diversity of assemblages harboured by mussel beds are related to location on the shore (i.e., tidal level), substratum and season. However, these factors were similar among sampling sites in our study so they should not be regarded as confounding factors. Hammond and Griffiths [36] showed that species richness and diversity were higher at the most exposed and most sheltered sites and lower at sites with intermediate wave exposure. In this sense, sampling sites in our study were similarly exposed to wave action and this factor is therefore not likely responsible for the observed differences.

Mussel size has been identified as the most important predictor for both abundance and species richness in natural mussel beds [27]. However, in our study values of these variables only showed significant differences between control and homogenised clumps in one out of the four studied sites. Furthermore, we would expect that mussels with dissimilar sizes would provide a more heterogeneous habitat (i.e., more variety of microhabitats and niches) than that provided by similar-sized mussels and, thus, a higher abundance and diversity of their associated assemblages [17, 47]. However, contrary to our expectations, homogenised clumps showed, in general, higher values in total number of individuals and species than control clumps. In agreement with our results, Singh et al. [9] found that a higher variability in mussel bed structure does not necessarily result in a higher number of species. O’Connor and Crowe [17] have pointed that in some studies the effect of mussel size is confounded with the age of mussel clumps [26]. As our study is observational, we are not able to separate the effects of clump age from mussel size homogenisation. However, patterns observed in our study did not seem to indicate an effect of clump age. If control clumps in our study represent older communities, according to the Ecological Time Hypothesis, communities that have existed for longer periods should have longer time for colonization and thus showing increased diversity [48]. However, we found generally a lower abundance for most of the species and diversity in homogenised clumps in comparison to control ones. Moreover, Tsuchiya and Nishihira [26] found greater diversity among large mussels, but their patch of large mussels was also older than the other examined patches. Our data, however, showed no significant differences in mussel length between control and homogenised clumps. Probably, if control clumps were older, they should have shown greater lengths than homogenised clumps. Moreover, O’Connor and Crowe [17] found an effect of mussel size in the structure of faunal assemblages in mussel clumps despite of being of similar age. For these reasons, patterns here reported do not seem to be conditioned by differences in the age between homogenised and control clumps.

Previous studies showed that mussel abundance [e.g., 12, 13, 23] and size [e.g., 26] were correlated with N and S of the associated assemblage. However, post-hoc analyses showed that differences in density and mean size of mussels followed a different pattern than that observed for N and S, which showed significantly higher values only in O2 whereas mussel density was significantly higher in both sites of Oia and for mean length no significant differences were found. Therefore, the density or size of mussels should not be related to observed differences in N and S in our study. Similar results were reported by Chintiriglou et al. [22] who found no relationship between mussel density and N and S.

Regarding the effect of mussel size homogenisation on the multivariate structure of the assemblages, we found significant differences between control and homogenised clumps in three of the four studied sites (i.e., M1, O1 and O2). Again, contrary to expectation, most taxa usually reached higher abundances in homogenised clumps than in control ones. Nematodes and oligochaetes (the latter only in O2) were among the taxa that contributed the most to dissimilarity between control and homogenised clumps, reaching much higher abundances in homogenised clumps. The presence of nematodes and oligochaetes are mostly linked to sediment [17], suggesting that homogenised clumps are able to retain more sediment than control clumps. In fact, the tighter cluster of mussels in homogenised clumps creates more suitable conditions to accumulate and preserve sediment because such tighter packing reduce the effects of wave impact [49] compared to control clumps; the latter are composed by mussels of different sizes, that form a less cohesive bed and are, in turn, more prone to wave washing, and hence, to sediment removal. Previous studies corroborated that the amount of sediment trapped in mussel clumps influences their associated faunal assemblages. For instance, Iwasaki [49] proposed that differences in community composition in beds of two mussel species resulted from changes in the sediment content trapped in clumps. Similarly, Prado and Castilla [50] found that in beds of *Perumytilus purpuratus*, sediment content shaped the community composition. Moreover, McQuaid and Dower [51] reported an increase in the number of species in rocky shores regularly flushed with sand. These patterns have been related to the increase of the habitat heterogeneity due to presence of sand. Likewise, Koivisto and Westerbom [52] showed that the sediment among mussels provides an important habitat for bottom dwelling fauna, while Lintas and Seed [53], studying the fauna associated with *Mytilus edulis*, found that occurrence of typical infaunal taxa, closely related to soft environments, depended on the presence of sediment within mussel beds.

In the same manner as nematodes, the bivalve *L*. *rubra* was among the most important taxa responsible for dissimilarities between assemblages associated with control and homogenised clumps. Lintas and Seed [53] suggested that this species is patchily distributed with marked differences in abundance depending on the site. However, in our study *L*. *rubra* always reached higher abundances in the homogenised clumps. Being a crevice-dwelling bivalve, it may benefit from the presence of sandy sediment inside mussel beds. In fact, *M*. *edulis* beds free of excessive silt content may harbour great abundances of *L*. *rubra* [54].

Other relevant species responsible for differences on assemblage structure between control and homogenised clumps were the amphipods *Hyale* spp. and *S*. *monoculoides*, and the isopod *J*. *albifrons*. These species reached higher abundances in homogenised clumps with the exception of M1, where they showed higher values in control clumps. These species graze mainly on algae or bacterial films [55–57] and therefore it is likely that food availability could play an important role controlling their abundance among mussel beds since they promote microvegetation growth such as cyanobacteria or diatoms [19] and even macroalgae [21]. In fact, previous studies have shown that the presence of the macroalga *Fucus vesiculosus* in mussel beds increased the abundance of mobile algal herbivores [52, 58]. In view of the abundance of these crustaceans, it is likely that in homogenised clumps there could be more available algae, allowing crustaceans to reach higher abundances at all locations, with the exception of M1. O’Connor and Crowe [17] also found that *Jaera forsmani* was more abundant in smaller mussels, probably by the presence of greater amounts of microalgae, and pointed out the importance of feeding preferences and availability of resources for mussel community structure. However, contrary to our results, O’Connor and Crowe [17] found that *Hyale prevosti* was equally distributed in different patches of larger and small mussels.

Another species responsible for differences between faunal assemblages in control and homogenised clumps was the gastropod *R*. *parva*, that followed the same general pattern as described above (i.e., higher abundance in homogenised clumps). In this sense, O’Connor and Crowe [17] also found that *R*. *parva* was more abundant when associated with smaller mussels. *Rissoa parva* is a detritivore usually associated to coralline or branched algae where diatoms and detritus get trapped [59]; previous work suggested that smaller mussels provide greater food availability for this species and their small size is enough to fit the space between small mussels [17]. Our results suggest that homogenised clumps seem therefore more suitable for this species.

Biotic interactions may also play an important role shaping the structure of assemblages associated with mussels [13, 23], although they have been rarely studied [22]. For instance, predation by sea otters in the Pacific results in mussels of smaller sizes (i.e., reduction in available habitat for fauna) [9]. Predation is also considered an important factor shaping the community structure and abundance of amphipod and isopod crustaceans, which are important food items for several species [57, 60]. According to ecological theory, predation should be a prevailing factor shaping the assemblage structure in mild environments [61–63]. However, in exposed mussel beds, such as those studied here, the influence of predation does not seem important enough to impact community structure [10].

Our study emphasizes the importance of *M*. *galloprovincialis* beds as providers of habitat because they harbour rich faunal assemblages. Indeed, the total number of species reported in our study (114) is near the highest reported elsewhere for fauna associated with *M*. *galloprovincialis* according to the table review in Chintiroglou et al. [22]. Therefore, the degradation of mussel beds will surely contribute to biodiversity loss in marine ecosystems. In this way, and as suggested in some previous studies [e.g., 64, 65], our results also demonstrate that ecosystem engineers should be considered important target species for conservation issues. Particularly, mussel beds could be useful indicators of hotspots with great biodiversity [47]. As mussels are important economic resources, policy regarding their management should also take into account their relevant ecological functions [16, 66]. Some studies have raised awareness of climate change and its consequences for the large biodiversity harboured by mussels [e.g., 34, 67]. However, recent work showed that mussels are mostly familiar to the general public just as food resource and encouraged for efforts to increase public awareness about the importance of mussel beds [68].

Our results also indicated that spatial variability interacts with the effect of habitat homogenisation on the structure of faunal assemblages harboured by mussels. Therefore, it is difficult to extrapolate our results to other localities and this could explain the contrasting results about the relationship between the mussel population structure and their associated biodiversity [12, 13, 22, 23, 26]. We also provide information about the species contribution to dissimilarities among homogenised and control clumps and discuss the potential influence of sediment and algae on mussel clumps. However, manipulative experiments are needed to determine whether the effect of spatial variability on mussel-associated fauna is indirectly linked to the habitat structure provided by them, particularly regarding the presence of sediment and algae, or whether observed effects are due to independent site effects on both variables (i.e., mussel and fauna). This will also serve to clarify the potential effect of biotic interactions occurring inside and outside of the mussel habitat.

## Supporting information

S1 Table Results of SIMPER analysis. Contribution (δi) of individual taxa to the average Bray-Curtis dissimilarity between treatments at the studied sites that showed significant differences in epifaunal assemblage structure.
(PDF)

S2 Table Full data. (XLSX)
