# Supplementary material for: Effects of habitat homogenisation on assemblages associated with mussel clumps
Source: PLoS One. 2022 Jun 1;17(6):e0269308. doi: 10.1371/journal.pone.0269308 (PMC9159611; doi:10.1371/journal.pone.0269308)
Supplement: S1 Table — Contribution (δi) of individual taxa to the average Bray-Curtis dissimilarity between treatments at the studied sites that showed significant differences in epifaunal assemblage structure. (PDF) [file pone.0269308.s001.pdf]

S1 Table.

Species	HT	M1 Average Abundance					O1 Average Abundance					O2 Average Abundance				
		Control	Homogenised	δ*i*	δ*i*%	δ*i*/SD(δ*i*)	Control	Homogenised	δ*i*	δ*i*%	δ*i*/SD(δ*i*)	Control	Homogenised	δ*i*	δ*i*%	δ*i*/SD(δ*i*)
Rissoa parva	M	16.25	45	9.12	17.17	1.74						14.25	68	3.04	4.58	0.98
Hyale spp. (juveniles)	C	24.25	0	6.99	13.17	2.24	16.75	42.75	8.44	14.24	1.36	20.75	44	1.39	2.08	1.53
Nematoda	O	50	61.75	6.51	12.26	1.62	9.75	58.75	16.57	27.94	1.84	73	375.5	16.17	24.33	2.26
Hyale nilssoni/stebbingi	C	11.25	0	3.39	6.39	2.17	5.75	12	2.55	4.3	1.19	11.25	16.25	0.6	0.9	1.88
Lasaea rubra	M	1	12.5	3.08	5.8	1.06	4.75	26.75	7.3	12.32	0.61	138.5	149.75	4.32	6.5	1.25
Skeneopsis planorbis	M	6.25	12	2.51	4.73	1.62						1.25	9.25	0.46	0.7	1.65
Nemertea	O	4.5	10.75	2.29	4.31	1.76	1.25	2.5	0.58	0.98	1.29					
Odostomia scalaris	M	7	9	1.94	3.64	1.11	8.75	1.75	2.53	4.27	1.44	19.5	72.5	3.16	4.76	1.32
Nucella lapillus	M	8	4.5	1.59	2.99	1.25										
Idotea pelagica	C	4.75	0	1.38	2.59	1.46	6.75	0.25	2.37	3.99	1.67					
Dolichopodidae n.i.	H	0.25	4.75	1.29	2.44	1.99										
Chironomidae n.i.	H	2.5	6.75	1.28	2.41	2.04	3	0.25	1.15	1.93	0.77	7.25	29.5	1.32	1.99	1.61
Gibbula umbilicalis	M	5.75	2.5	1.28	2.4	1.14						16	9.5	0.86	1.29	1.69
Syllis vivipara	A	0	4.5	1.26	2.37	0.86										
Stenothoe monoculoides	C	4	0	1.17	2.21	1.25						17.25	136.25	6.47	9.73	2.49
Jaera albifrons	C	2.75	0	0.79	1.48	0.93	11.25	25.75	7.59	12.8	1.17	10.75	22	0.85	1.27	1.11
Campecopea lusitanica	C	2.5	0	0.66	1.24	0.72										
Actina equina	N	2	1.75	0.65	1.22	1.21										
Eulalia clavigera	A	1.25	1.75	0.54	1.01	1.2										
Barleia unifasciata	M	0.75	1.75	0.4	0.76	1.26						3	31.25	1.64	2.46	1.51
Harpacticoida n.i.	C						1.75	6	1.65	2.78	1.26	8.75	27	1.05	1.58	1.67
Patella depressa	M						6.25	7	1.53	2.57	1.19					
Hyale perieri	C						2.25	3	0.9	1.52	1.22					
Ischyromene lacazei	C						5.25	3.5	0.63	1.07	1.28					
Oligochaeta n.i.	A											2.75	262	13.69	20.59	1.63

HT: higher taxon; A: Annelida; C: Crustacea; H: Hexapoda; M: Mollusca; N: Cnidaria; n.i.: not identified to species level; O: Other.
